# Action at a distance: dependency sensitivity in a New World primate

**DOI:** 10.1098/rsbl.2013.0852

**Published:** 2013-12-23

**Authors:** Andrea Ravignani, Ruth-Sophie Sonnweber, Nina Stobbe, W. Tecumseh Fitch

**Affiliations:** Department of Cognitive Biology, University of Vienna, Althanstrasse, 14, Vienna 1090, Austria

**Keywords:** language, music, New World monkey, computation, perception, pattern

## Abstract

Sensitivity to dependencies (correspondences between distant items) in sensory stimuli plays a crucial role in human music and language. Here, we show that squirrel monkeys (*Saimiri sciureus*) can detect abstract, non-adjacent dependencies in auditory stimuli. Monkeys discriminated between tone sequences containing a dependency and those lacking it, and generalized to previously unheard pitch classes and novel dependency distances. This constitutes the first pattern learning study where artificial stimuli were designed with the species' communication system in mind. These results suggest that the ability to recognize dependencies represents a capability that had already evolved in humans’ last common ancestor with squirrel monkeys, and perhaps before.

## 1. Introduction

Human language relies on several basic and indispensable cognitive skills, including the detection of relationships or ‘dependencies’ between stimuli that are non-contiguous in space or time. Dependency sensitivity, defined here as the ability to recognize that two non-contiguous sensory items are related (e.g. belong to the same perceptual class), is part of everyday sensory experience and crucial for many aspects of human cognition [1–3].

The perceived ‘musicality’ of some languages results from how syllable types are combined to form words. In Turkish, for instance, the plural of a noun is formed by adding a suffix to its singular form. Crucially, the suffix's vowel must belong to the same *acoustic class* as the noun's last vowel, hence establishing an abstract dependency (not between specific items). Hungarian, like Turkish, also exhibits such ‘vowel harmony’. In Hungarian, the first and last vowels depend on each other but they can be separated by several neutral syllables, thus exhibiting an arbitrary-distance dependency between non-adjacent elements.

Dependencies that are *both* abstract (applying to classes of elements) and occur at variable distance are essential in productively open systems like language and music. The evolutionary origins, e.g. in primates, of the cognitive ability to detect dependencies are unknown. Human infants already possess the capacity to track non-adjacent dependencies in natural language [3]. In ‘artificial languages’, dependencies between non-adjacent elements are particularly easy to detect if occurring between perceptually similar elements [2,4] or at the edges of stimuli [5].

Previous comparative animal research has demonstrated awareness of dependencies *either* occurring at a *fixed distance* [6,7] or between *specific items* [5]. Detection of abstract dependencies at arbitrary variable distances (crucially beyond one intervening element, already shown in [4,7]) has never been demonstrated before in a non-human animal (though see [8] for initial hints). The current study tested the hypothesis that a non-human primate species could detect abstract, non-adjacent dependencies in acoustic stimuli, even when dependencies occurred over an arbitrary variable number of intervening sounds.

**Figure 1.**
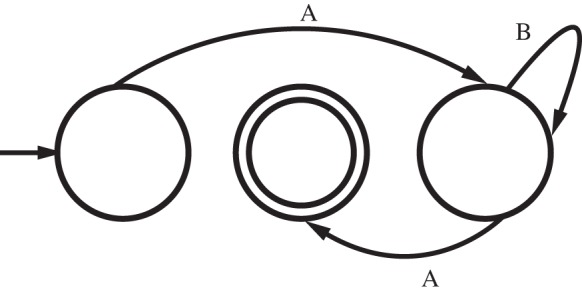
Finite state machine generating and recognizing AB*^n^*A strings. Every transition (arrows) from one state to another (circles) produces a new element of the string (A or B). Any sequence of transitions beginning in the (leftmost) start state (denoted with an arrow) and finalizing in the accept state (denoted by concentric circles) produces a string containing a dependency.

We used formal language theory as a precise mathematical framework to characterize string complexity [9,10]. The formal language used to generate stimuli [11], AB*^n^*A (not employed in empirical research before) captures a single arbitrary-distance dependency between similar elements at its edges (figure 1). AB*^n^*A characterizes strings with one A at the beginning, one A at the end, and *n* repetitions of B in between. Any other combination of As and Bs violates this rule. Notably, this pattern captures aspects of naturally occurring linguistic phenomena (as seen for Hungarian), while taking into account edge and perceptual similarity effects in designing the stimuli [4,5].

## 2. Material and methods

### (a) Subjects and experimental procedure

Six group-housed squirrel monkeys (*Saimiri sciuresu*) were individually trained over eight months to enter a sound booth voluntarily. The experiment used a habituation–discrimination paradigm [5], consisting of exposure to habituation stimuli and individual testing using novel stimuli. If able to perceive the relation of dependency between the first and last A elements, monkeys should react differently when tested with sounds obeying, versus those violating, the rule.

Animals were habituated to 360 stimuli (two sessions over 2 days), played in random order to all individuals simultaneously (80 min total).

During the test phase, individual monkeys entered the acoustic booth and sat on a perch. One experimenter inside the booth (wearing headphones playing custom-generated masking music to prevent unconscious cuing) fed insects to the subject *between* playbacks.

### (b) Stimuli description

Exposure and test stimuli were generated following the AB*^n^*A rule. As and Bs were mapped to two pure sine wave tone classes, high (H) and low (L), consisting of 44 elements each. ‘Low’ tone frequencies were randomly and uniformly sampled from an interval centred at 2 kHz; ‘High’ tones had mean frequency 11 kHz (interval endpoints: ±10% of mean; duration: 225 ± 15 ms). For the habituation, As were matched to the low category and Bs to the high category, (*n* = 1,…,3). Thus, monkeys were habituated to a set of three patterns: LHL, LHHL and LHHHL.

The frequency classes here were chosen because: (i) squirrel monkeys are equally sensitive to sounds in these frequency ranges [12], (ii) durations and frequencies of species specific vocalizations exist in these ranges [13] and (iii) pure tones avoid potential confounds involved in using recorded monkey calls, where reactions might be elicited by the meaning attached to calls, rather than patterns formed from them. Furthermore, inspired by the time-domain characteristics of squirrel monkeys’ vocalizations [13], the tones composing our stimuli are markedly shorter than the units employed in previous similar experiments. To our knowledge, this is the first animal pattern perception experiment using pure tone stimuli specifically tailored to a particular species’ communication system.

### (c) Video coding and data analysis

We eliminated the possibility of coder bias with three concurrent coding strategies: (i) reactions were videotaped and coded by multiple raters, who were (ii) otherwise not involved in this experiment, and blind to the hypothesis being tested and (iii) completely blind to which stimulus was played [14], to ensure that no bias could affect coding decisions. Our method [14] involves replacing the original audio of the experiment with sinewave placeholders, ruling out knowledge of which stimulus was played.

Three colleagues annotated head turns towards the loudspeaker of 45° or more. Before video coding started, we established the criterion that only head turns starting after stimulus onset and within 7 s from the playback onset (four times the duration of the longest stimulus) would be extracted from the annotations and further analysed. The average index of concordance [15] was 0.875 (calculated on 24 trials unused in this study).

Data analysis was performed in SPSS and STATA. Parametric tests were used after testing for normality (Shapiro–Wilk) and homoskedasticity (Levene) (*n* = 6 or *n* = 4, all *p*-values ≥ 0.27).

### (d) Test 1

Test 1 investigated whether squirrel monkeys (i) acquired the dependency rule, showing different reactions between stimuli obeying or violating it, (ii) generalized the rule over new instantiations of sound patterns and (iii) generalized to dependencies between low sounds separated by a previously unheard number of intervening high sounds (extensions).

Half the stimuli for test 1 were *consistent* with the exposure rule (C_1_, index indicating test 1) and half represented *violations* (V_1_) of the dependency rule (table 1). Consistent stimuli either followed the same overall pattern and length as habituation stimuli, but involved novel tone combinations (the particular tones composing each pattern were re-sampled anew from their respective pitch classes) or contained a previously unheard number of intervening low tones, generalizing the rule by induction over *n*.

**Table 1.** Experimental patterns. Breakdown of stimuli type by class and subclass, and number (specified when greater than 1) of different exemplars the monkeys were exposed to during the habituation and the tests.

stimulus class	subclass	Test 1	Test 2
habituation		LHL (60), LH^2^L (120), LH^3^L (180)	
consistent	repetition	LHL, LH^2^L, LH^3^L (2)	HLH, HL^2^H, HL^3^H (2)
	extension	LH^4^L (2), LH^5^L (2)	HL^4^H (2), HL^5^H (2)
violation	missing first	HL, H^2^L, H^3^L, H^4^L	LH, L^2^H, L^3^H, L^4^H
	missing last	LH, LH^2^, LH^3^, LH^4^	HL, HL^2^, HL^3^, HL^4^

### (e) Test 2: meta-generalization

Before this test, no novel habituation stimuli were presented. The only difference between test 2 and test 1 was that the mapping between low and high tones was inverted, so that in test 2 As corresponded to high tones and Bs to low frequencies (e.g. HLH).

A monkey succeeding at test 2 should perceive a habituation stimulus like LHHL and a test stimulus, like HLLLH as belonging to the same class, while regard a sound such as HLLL as a violation to the original rule LH*^n^*L.

## 3. Results

**Figure 2.**
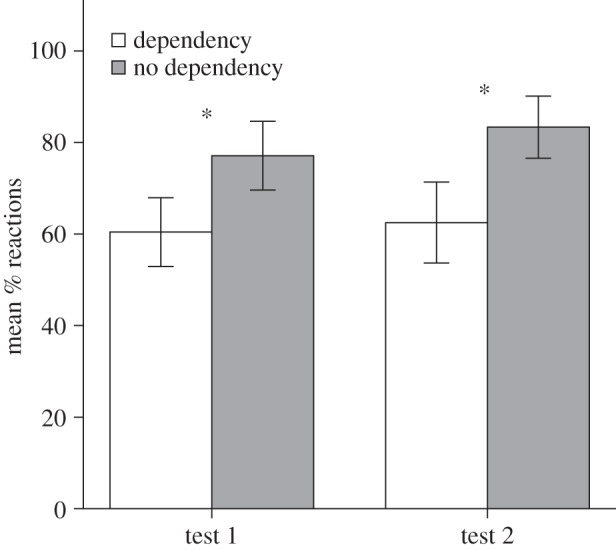
Histograms for percentage reactions in test 1 (left, *n* = 6) and test 2 (right, *n* = 4). The average percentage consistent (white) and violation (grey) trials that elicited a reaction are displayed in each case (mean ± s.e.m.). For test 2, reactions to novel violations (see Results) are shown.

For each monkey, PR(V_1_) was greater than or equal to PR(C_1_) (PR = percentage of reactions), with PR(C_1_) = 60.4% and PR(V_1_) = 77.1% (s.d.: 18.4 both). Overall, PR(V_1_) differed significantly from PR(C_1_) (figure 2; paired *t*-test, *n* = 6, *t* = 3.16, *p* = 0.025). Responses did not differ between stimuli missing the first or last low tone (*n* = 6, *t* = 0.54, *p* = 0.611; see electronic supplementary material, S1).

In test 2, the monkeys did not show any difference between PR(C_2_) and PR(V_2_). (paired *t*-test, *n* = 4, *t* = 1.98, *p* = 0.141.) Taking test 2 after test 1 might have generated order effects (monkeys could have habituated to two violation stimuli, HL and LH, presented in test 1, see electronic supplementary material, S1). In fact, a paired *t*-test, comparing PR(C_2_) to PR(V’_1_) (novel violations) showed a significant effect of stimulus type on response (figure 2, *n* = 4, *t* = 4.64, *p* = 0.019), suggesting a generalization from LH*^n^*L to HL*^n^*H.

We ran a repeated measures ANOVA involving test type (test 1 versus test 2) and grammaticality (violation versus consistent). Reactions to LH and HL were also excluded in test 1, to maintain a one-to-one correspondence between stimuli across tests. We found an effect of grammaticality (2 × 2 ANOVA, *n* = 4, *F* = 23.14, *p* = 0.017); but no effect of test type (*F* = 0.06, *p* = 0.822) and no interactions (*F* = 0.27, *p* = 0.638).

## 4. Discussion

Squirrel monkeys consistently recognized and generalized the pattern AB*^n^*A at different levels, showing sensitivity to arbitrary-distance dependencies.

Test 1 showed that our subjects effectively generalized the specific pattern beyond specific pitches or stimulus lengths. Rather than matching specific pitches, the monkeys attended to *relations* between sound categories when discriminating between stimuli containing or lacking a dependency. Together, both tests suggest that generalization to a higher level of abstraction, featuring previously unseen combinations of elements, occurred based solely on specific instantiations of the sound classes heard during the exposure. We were able to rule out some alternative, lower level explanations through our design and additional tests (e.g. monkeys do not attend exclusively to one of the stimulus' edges, see electronic supplementary material, S1): testing primates in an operant setup could help exclude additional simpler discrimination strategies.

Previous animal research has dealt mainly with dependencies occurring at a fixed distance: namely, at no more than one element apart. The formal language AB*^n^*A we used has relatively low computational complexity (finite state, strictly three-local [9]), but nonetheless possesses adequate representational power to capture dependencies between elements at arbitrary distance. In fact, the presence of sensory dependencies and grammar complexity can be orthogonal questions. Previous experiments whose stimuli included the AB*^n^*A substring do not provide evidence of dependency processing: super-grammars featuring AB*^n^*A can be mastered (significantly) without processing dependencies, and vice versa.

Pattern perception experiments aim to test cognitive abilities involving high-level properties of the patterns, rather than basic acoustic perception skills or semantic biases [10]. Many previous studies used human speech syllables, which may not be salient to all animal species. Pilot work with patterns made up of human syllables indicated a lack of discrimination between stimuli classes: our short high-frequency tone units might have enhanced performance.

Squirrel monkeys are sensitive to abstract dependencies of different lengths and can generalize to new lengths and auditory parameters of the stimuli. Human and squirrel monkey lineages diverged at least 36 Ma [16], and our findings suggest that dependency sensitivity was present in these primate ancestors. If so, most living apes and monkeys should exhibit this ability, which need not be evolutionarily related to communication and vocal flexibility, but could be a by-product of other cognitive abilities.

Despite its value in both language and music, dependency sensitivity apparently did not evolve specifically for use in these cognitive systems. Although no squirrel monkey will probably ever speak a human language, these monkeys possess the cognitive potential to recognize the rule generating plurals of Turkish nouns, or many other linguistic phenomena.

## 

Experimental procedures were non-invasive and in accordance with Austrian legislation.
